# Molecular Characterization and In Silico Functional Insights into Carbapenem Resistance in Clinical *Klebsiella pneumoniae* Isolates from Al-Diwaniyah, Iraq

**DOI:** 10.3390/pathogens15080819

**Published:** 2026-08-03

**Authors:** Nada Ahmed Fairooz, Amal Ben Hassena, Baheega Abees Al Khalidi, Erdi Can Aytar, Mohamed Sami Aifa, Mounira Hmani

**Affiliations:** 1 Diwaniyah Technical Institute, AL-Furat AL-Awsat Technical University, Najaf 54003, Iraq; 2 Laboratory of Electrochemistry and Environment, National School of Engineers of Sfax, University of Sfax, BP 1173, Sfax 3038, Tunisia; 3 College of Nursing, University of Al-Qadisiyah, Al-Diwaniyah 58001, Iraq; baheeja.alkhalidi@qu.edu.iq; 4 Department of Horticulture, Faculty of Agriculture, Usak University, Uşak 64200, Türkiye; erdicanaytar@gmail.com; 5 Laboratory of Molecular and Cellular Screening Processes, Centre of Biotechnology of Sfax, Sfax 3018, Tunisia; msamiaifa@gmail.com; 6 Molecular and Functional Genetics Laboratory, Faculty of Science of Sfax, University of Sfax, Sfax 3000, Tunisia; mounirahmani@gmail.com

**Keywords:** *Klebsiella pneumoniae*, XDR, MDR, antimicrobial resistance, carbapenem-resistant, carbapenemase genes, mutation analysis, genetic diversity, Al-Diwaniyah, Iraq

## Abstract

**Background**: The spread of extended-spectrum β-lactamase-producing *Klebsiella* is an emerging public health concern presenting severe clinical impacts. This problem is particularly severe in developing countries where irrational use of antibiotics makes the treatment of such infections more challenging. **Methods**: In this cross-sectional study, we investigated antibiotic resistance among clinical *Klebsiella pneumoniae* isolates from 256 patient specimens from Al-Diwaniyah hospitals, using standard microbiological methods followed by PCR. Antimicrobial susceptibility testing identified resistance rates and proportions of multidrug-resistant (MDR) and extensively drug-resistant (XDR) strains. Carbapenemase genes were detected by multiplex PCR and sequencing. Mutations were characterized, and their functional significance was predicted using in silico prediction tools. **Results**: Fifty *Klebsiella pneumoniae* isolates were recovered, exhibiting high resistance rates (42–100%) to penicillins, cephalosporins, fluoroquinolones and carbapenems, with 62% classified as MDR and 38% as XDR. Carbapenemase genes were highly prevalent (*blaOXA-48* 56%, *blaIMP* 44%, *blaVIM* 30%, *blaKPC* 28%, *blaNDM* 26%), with 60% of isolates co-harbouring ≥ two genes. Most mutations were predicted to be structurally tolerated, while active-site-proximal substitutions (H120L in NDM-52 and V120L in OXA-48) were predicted to affect enzyme activity. However, docking analysis suggested no significant alteration in carbapenem binding affinity. **Conclusions**: Our results highlight a very high prevalence of MDR/XDR *Klebsiella pneumoniae*, associated with diverse carbapenemase genes and resistance-related polymorphisms that may indicate potential functional impacts. To the best of our knowledge, this is the first study in Iraq to combine carbapenemase gene mutation analysis with structural modelling and molecular docking. This study emphasizes the urgent need for effective antimicrobial resistance surveillance in Iraq.

## 1. Introduction

*Klebsiella pneumoniae* is a Gram-negative, encapsulated, non-motile bacterium belonging to the *Enterobacterales* family. It is known as one of the most important opportunistic bacterial pathogens responsible for infections acquired both in the community and in healthcare settings [1,2,3]. This species is associated with a wide spectrum of diseases, including urinary tract infections, pneumonia, bacteremia, meningitis, wound infections and purulent abscesses [4,5]. *K. pneumoniae* can also be responsible for severe disseminated infections such as pyogenic liver abscesses, osteomyelitis and endophthalmitis, even in generally young and healthy individuals [6,7]. Furthermore, multidrug-resistant *K. pneumoniae* strains can cause treatment failure with current antibiotic therapies [8,9]. In fact, the rapid emergence of the extended-spectrum β-lactamase (ESBL) producing *K. pneumoniae* has significantly increased the risk of serious community-acquired infections worldwide [10] and poses a major threat to healthcare systems [11,12,13,14].

Carbapenems are a major class of β-lactam antibiotics with broad activity against Gram-negative bacteria and remain essential therapeutic options for severe infections caused by multidrug-resistant organisms. Although newer antimicrobial agents have become available for the management of specific resistance profiles, carbapenems continue to play a central role in the treatment of serious infections caused by Gram-negative bacteria [9,10,11,12,13,14,15]. However, the global emergence of carbapenem resistance has become a growing public health threat [16]. This resistance is becoming a serious concern because these strains are able to survive in multiple environments. Several mechanisms contribute to carbapenem resistance, such as modifications in outer membrane permeability due to the loss of porins, increased efflux systems and the overproduction of AmpC β-lactamases or extended-spectrum β-lactamases (ESBLs), or, more frequently, the synthesis of carbapenemases [17]. Among the most prevalent resistance mechanisms within *K. pneumoniae* against different classes of antibiotics is the activation of efflux pump systems and the release of antibiotic-inactivating enzymes [18]. Carbapenemase-encoding genes are now widespread in many regions globally [16]. The predominant gene is *Klebsiella pneumoniae* carbapenemase, KPC, and its quick international emergence has made it a recognized global public health concern [19,20,21]. Several other carbapenemases encoded by a heterogeneous family of genes are also widespread, among which the most prevalent are *blaNDM*, *blaOXA-48*, *blaIMP* and *blaVIM* [22]. The specific frequencies of such carbapenemase genes vary across populations [23,24]. A recent review from Iraq highlighted the regional diversity in carbapenemase genes, with the domination of *blaOXA-48* and *blaNDM-1* genes in several regions, while other genes such as *blaVIM*, *blaGES*, and *blaOXA-1* were reported at high rates in specific regions [25]. With an unfavourable history of indiscriminate antibiotic use in hospital and home-care settings, Iraq represents fertile ground for MDR *K. pneumoniae*. Moreover, given the limited studies on β-lactamase genes in the Middle Euphrates region of Iraq (Al-Diwaniyah, Najaf, Karbala, and Samawah), a comprehensive investigation into the prevalence of MDR *Klebsiella pneumoniae* strains and their β-lactamase genes is needed. In addition, there is currently no in-depth study investigating the capacity of these bacteria to develop mutations at the molecular level.

Therefore, this study aims to determine phenotypic resistance rates and MDR/XDR prevalence among clinical *K. pneumoniae* from Al-Diwaniyah; identify carbapenemase genes by multiplex PCR/sequencing; and characterize mutations in carbapenemase enzymes via in silico functional analysis and molecular docking.

## 2. Methods

### 2.1. Study Design and Sampling

This study is a cross-sectional observational study conducted on clinical samples collected from patients attending three hospitals in the Al-Diwaniyah Governorate, Iraq, between October 2021 and February 2022. This was an observational study with no longitudinal follow-up or patient-level clinical analysis. Bacterial strains were isolated from patients with suspected bacterial infections using various clinical specimens (urine, blood culture, high vaginal swab, sputum, ear swab, nasal swab, peritoneal fluid, urethral swab, pus swab, pleural fluid/aspirate and cerebrospinal fluid). Samples were collected from 256 patients in three Al-Diwaniyah hospitals in Iraq (Al-Diwaniyah Teaching Hospital, Al-Diwaniyah Chest center Hospital and the Maternity and Children’s Hospital) during the period from October 2021 to February 2022. Specimens were collected from hospitalized patients with suspected bacterial infections who were undergoing routine microbiological investigations requested by the attending clinicians. As this study focused on the microbiological characterization of clinical isolates, detailed patient-level clinical information and follow-up data were not collected. Only cultures yielding a single bacterial species were included. Samples with evident contamination, duplicate isolates from the same patient or insufficient volume were excluded from the study.

### 2.2. Phenotypic and Molecular Identification of Klebsiella pneumoniae Isolates

The isolates were cultured and characterized using standard microbiological and biochemical methods followed by identification with the VITEK 2 compact system (bioMérieux, Marcy-l’Étoile, France). Genomic DNA was extracted using the Presto™ Mini gDNA Bacteria Kit following the manufacturer’s instructions (Geneaid Biotech Ltd., New Taipei City, Taiwan). Molecular identification of *Klebsiella pneumoniae* was performed by PCR targeting the 16S rRNA diagnostic gene. The primers yielded an amplicon of 551 bp with the forward sequence F-CCTGGACAAAGACTGACGCT and the reverse sequence R-AGTTGCAGACTCCAATCCGG.

### 2.3. Evaluation of Antimicrobial Resistance in Klebsiella pneumoniae

#### 2.3.1. Antimicrobial Susceptibility Testing

The disk diffusion method was performed to assess the antimicrobial susceptibility of the isolated *Klebsiella* strains using the Kirby–Bauer method [26] on Mueller–Hinton agar (bioMérieux, Marcy-l’Étoile, France) with commercially available antibiotic disks (Oxoid Ltd., Basingstoke, Hampshire, UK). The antibiotics tested (and their disk contents) included ampicillin (25 μg), temocillin (30 μg), piperacillin/tazobactam (110/10 μg), meropenem (10 μg), imipenem (10 μg), ertapenem (10 μg), cefoxitin (30 μg), cefuroxime (30 μg), ceftriaxone (30 μg), ceftazidime (85 μg), cefotaxime (30 μg), cefepime (30 μg) and ciprofloxacin (5 μg). Protocols and interpretations of susceptibility (resistant, intermediate or susceptible) based on inhibition zone diameters were conducted according to the guidelines of the Clinical Laboratory Standards Institute (CLSI). Multidrug-resistant (MDR) and extensively drug-resistant (XDR) phenotypes were defined according to the standardized international consensus criteria, based on non-susceptibility to at least one agent in three or more antimicrobial categories for MDR, and non-susceptibility to at least one agent in all but two or fewer categories for XDR.

#### 2.3.2. Molecular Investigation of Carbapenemase Production

Genes associated with antibiotic resistance were amplified by conventional multiplex PCR using gene-specific primers. The latter were designed using the online primer design tool Primer-Blast, available through the National Center for Biotechnology Information (NCBI) https://www.ncbi.nlm.nih.gov/tools/primer-blast/, accessed on 1 July 2025. Table 1 provides a summary of primer pair sequences and PCR product sizes. PCR amplifications were performed in a total reaction volume of 50 µL, containing 5 µL of DNA template (5–50 ng), 1 µL of each forward primer (*blaKPC*, *blaIMP1*, *blaIMP2*, *blaVIM*, *blaNDM*, and *blaOXA-48*; 10 pmol each), and 1 µL of the corresponding reverse primers for each target gene (10 pmol each). The reaction mixture also included 25 µL of GoTaq^®^ Green PCR Master Mix (Promega Corporation, Madison, WI, USA) and 8 µL of ultra-pure water. The PCR protocol was determined using the Optimase Protocol Writer™ online application (Transgenomic, Inc., Omaha, NE, USA). PCR conditions were set as follows: an initial denaturation step for five minutes at 95 °C, followed by 35 cycles of three steps—30 s of denaturation at 95 °C, 30 s of annealing at 58 °C, and 1 min of extension at 72 °C. A final extension step was conducted for five minutes at 72 °C and the reaction was finally maintained at 4 °C. The amplified PCR products were analyzed by 1.5% agarose gel electrophoresis. DNA sequencing was performed using the Sanger method on an Applied Biosystems DNA sequencing platform.

#### 2.3.3. Identification of Nucleotide and Amino Acid Variations in Carbapenemase Genes

The partial sequences from the carbapenemase genes (IMP, NDM, KPC, VIM, and OXA families) were aligned to close homologs from the NCBI database using BLAST [27] (National Center for Biotechnology Information (NCBI), https://blast.ncbi.nlm.nih.gov/Blast.cgi, accessed on 1 July 2025). Nucleotide substitutions were identified by comparing each partial gene sequence to its closest reference. The corresponding amino acid changes were then determined by translating the DNA sequences using ExPASy [28] (Swiss Institute of Bioinformatics (SIB), Lausanne, Switzerland; https://web.expasy.org/translate/, accessed on 1 July 2025)and aligning the amino acid sequences against reference genes (retrieved from NCBI) using BLASTp [27] (National Center for Biotechnology Information (NCBI), https://blast.ncbi.nlm.nih.gov/Blast.cgi?PROGRAM=blastp&PAGE_TYPE=BlastSearch&LINK_LOC=blasthome, accessed on 1 July 2025. Each mutation was classified as ‘silent’ when the nucleotide change did not result in an amino acid change and as ‘missense’ when it resulted in an amino acid substitution.

#### 2.3.4. Computational Assessment of Functional and Structural Impacts of Point Mutations on Carbapenemases

The structural and functional effects of point mutations in carbapenemase enzymes were evaluated using a combination of sequence- and structure-based computational methods. Missense amino acid substitutions were first analyzed with the PROVEAN (Protein Variation Effect Analyzer, developed at J. Craig Venter Institute (JCVI), Rockville, MD, USA, http://provean.jcvi.org, accessed on 1 July 2025, and SIFT (Sorting Intolerant From Tolerant) algorithms, developed at Fred Hutchinson Cancer Research Center, Seattle, WA, USA, https://sift.bii.a-star.edu.sg/, accessed on 1 July 2025. PROVEAN provides a functional impact score based on sequence conservation and alignment. SIFT evaluates the functional impacts of amino acid substitutions. The SIFT tool predicts the effect of amino acid substitutions on protein function based on a score classifying mutations as ‘deleterious’ (≤0.05), indicating a high probability of functional impact (mutations located near the active site), and ‘tolerated’ (neutral), for those with a score > 0.05. Such analysis suggests potential alteration of substrate hydrolysis or structural stability of the enzyme. A PROVEAN score under −2.5 defines the mutation as deleterious and a score > −2.5 considers it as neutral/tolerated (with no impact on function).

The DDMut tool was further used to study protein stability modifications (Bio-Sig Laboratory, The University of Queensland, Brisbane, QLD, Australia; http://biosig.lab.uq.edu.au/ddmut/, accessed on 1 July 2025). It predicts how point mutations affect protein stability throughout the prediction of Gibbs free energy variations (ΔΔG). A negative ΔΔG designates a destabilizing mutation that might enhance the protein flexibility and improve conformational adaptation or decrease enzymatic function. A positive ΔΔG indicates a stabilizing mutation that could improve structural integrity or reduce the flexibility of the catalytic function. For the DDMut tool, an absolute ΔΔG > 1 kcal/mol indicates significant alteration.

The FiTMuSiC platform uses structural and evolutionary data to assess the impact of mutations on protein stability and function (Université Libre de Bruxelles [ULB], Brussels, Belgium; http://babylone.ulb.ac.be/FiTMuSiC/), accessed on 1 July 2025. FiTMuSiC classifies mutations as neutral, gain of function, partial loss or deleterious. FiTMuSiC scores near 1 denote neutral effects and values above 1 suggest potential gain of function, while scores between 0 and 1 indicate partial loss, and scores ≤ 0 indicate deleterious mutations. Negative z-scores signify functional or structural loss and positive values indicate tolerated mutations. Overall, a mutation is considered neutral if |Z| ≤ 1, has a moderate effect if 1 < |Z| ≤ 2, and is predicted to have a strong effect if |Z| > 2. Structural (Struct Z) and evolutionary conservation (Evol Z) z-scores give additional characterization for mutations; negative Struct Z and Evol Z scores reflect structural destabilization and evolutionarily destabilizing alteration, respectively.

#### 2.3.5. Protein Preparation and Mutagenesis

The three-dimensional structures of the wild-type carbapenemases were retrieved from the Protein Data Bank (PDB): IMP-1 (PDB ID: 1JJT), IMP-2 (PDB ID: 4UBQ), VIM-1 (PDB ID: 5N5I), KPC-2 (PDB ID: 5UL8), OXA-48 (PDB ID: 5DTK) and NDM-1 (PDB ID: 5YPL). Protein preparation and mutagenesis were performed using Discovery Studio (BIOVIA Discovery Studio Visualizer, version 2017 (BIOVIA, San Diego, CA, USA)). The 3D structures were cleaned to retain one protein chain and to eliminate any alternate conformations, while Zn^2+^ ions in metallo-β-lactamases, as well as native ligands within the active sites, were preserved. Using Discovery Studio, in silico site-directed mutagenesis was then performed with the ‘build and edit protein’ and ‘Mutate’ tools to replace target residues with specific amino acid substitutions. Subsequently, the geometry of the structures was corrected. The final wild-type and mutant protein chains were saved in PDB format for subsequent molecular docking analysis.

#### 2.3.6. Molecular Docking Analysis of Wild-Type and Mutant Protein Chains

In order to assess whether the amino acid mutations identified in carbapenemase sequences affect the binding of their natural substrate, molecular docking analysis was performed. Meropenem was chosen as the substrate (ligand) due to its recognition by all tested genes. The ligand was drawn in two dimensions using ChemDraw Professional 16.0 software and subsequently converted to a three-dimensional conformation with Chem3D 16.0 (PerkinElmer, Waltham, MA, USA), followed by geometric optimization. The binding affinities of the ligand to the active sites of the target proteins were calculated using AutoDock Vina version 1.2.3) [29]. The grid box parameters were defined individually for each protein target, with center coordinates set as follows: IMP-1 (57, 24, 32), IMP-2 (5, 18, 29), VIM-1 (22, 13, 52), KPC-2 (16, 9, 20), OXA-48 (5, 32, 154), and NDM-1 (−41, 76, 93). The grid box dimensions were set to 23 × 23 × 23 Å for all protein structures. AutoDock Vina calculations were performed using default scoring parameters, with an exhaustiveness value of 8 and a maximum number of generated binding poses (num_modes) set to 9. Molegro Virtual Docker version 6.0 (Aarhus, Denmark) was used to explore alternative binding modes, validate results through additional scoring functions, and perform detailed structural analysis of the binding sites. The docking results were visualized in three dimensions using Discovery Studio version 2021 (BIOVIA), enabling comprehensive evaluation of binding modes and characterization of molecular interactions such as hydrogen bonds, hydrophobic contacts, and π-π interactions. Differences in binding affinity values between mutant and reference proteins were used to evaluate possible functional modifications due to amino acid substitutions. A binding score difference greater than 1 kcal/mol is considered as significant.

### 2.4. Statistical Analysis

Resistance rates, multidrug-resistant (MDR) and extensively drug-resistant (XDR) classifications, and gene prevalence were calculated as counts and percentages using Microsoft Excel 2016. As this was a descriptive cross-sectional study, no inferential statistical analyses were performed. Furthermore, the relatively limited number of isolates and the diversity of carbapenemase gene combinations limited the feasibility of making robust statistical comparisons between resistance phenotypes and gene carriage. Computational predictions of protein variants were performed using PROVEAN, SIFT, and DDmut according to their default recommended thresholds.

## 3. Results

### 3.1. Detection and Identification of Klebsiella pneumoniae Isolates

In the current study, a total of 256 clinical samples were collected between October 2021 and February 2022 from patients presenting to three hospitals in the Al-Diwaniyah Governorate, Iraq. The distribution of samples was as follows: urine (68, 26.56%), blood culture (39, 15.23%), sputum (30, 11.72%), ear swab (23, 8.98%), high vaginal swab (20, 7.81%), pus swab (20, 7.81%), urethral swab (18, 7.03%), nasal swab (15, 5.86%), peritoneal fluid (10, 3.91%), cerebrospinal fluid (eight, 3.13%), and pleural fluid/aspirate (five, 1.95%).

All *Klebsiella pneumoniae* isolates were initially characterized based on culture morphology and standard microbiological methods. On MacConkey agar, colonies appeared large, mucoid, and pink, indicating lactose fermentation, while on blood agar, they were greyish-white, mucoid, and non-hemolytic. Gram staining revealed Gram-negative rods occurring singly or in pairs, consistent with *Klebsiella* spp. Biochemical profiling showed typical reactions for *K. pneumoniae*, including oxidase negativity, catalase positivity, citrate utilization, and a characteristic IMViC pattern (indole negative, methyl red negative, Voges–Proskauer positive).

Final confirmation was obtained using the VITEK 2 Compact, which identified the isolates as *K. pneumoniae* with high confidence (95–99%). Molecular confirmation by PCR targeting the 16S rRNA gene showed that all isolates (100%) produced the expected 551 bp amplicon (Supplementary Figure S1).

Overall, fifty *Klebsiella pneumoniae* isolates were recovered, with a frequency of 19.53% (50/256). The distribution of isolates according to the source of infection was as follows: urine (14, 28%), blood culture (nine, 18%), high vaginal swab (nine, 18%), sputum (six, 12%), ear swab (four, 8%), nasal swab (three, 6%), peritoneal fluid (one, 2%), urethral swab (one, 2%), pus swab (one, 2%), cerebrospinal fluid (one, 2%) and pleural fluid/aspirate (one, 2%).

### 3.2. Resistance Rates Within Clinical K. pneumoniae Strains

The results of the antibiotic resistance rates for the 50 *K. pneumoniae* strains are summarized in Table 2. *K. pneumoniae* isolates showed very high resistance to penicillin antibiotics, with 100% resistance to ampicillin and 94% for temocillin. In total, 90% of the strains exhibited phenotypic resistance to second-generation cephalosporins, specifically cefoxitin and cefuroxime. For third-generation cephalosporins, resistance rates were 100% for cefotaxime, 96% for ceftriaxone and 90% for ceftazidime. In addition, a large proportion of the isolates (80%) exhibited resistance to the fourth-generation cephalosporin cefepime. For fluoroquinolones, 58% of the isolates showed decreased susceptibility to ciprofloxacin. Regarding antibiotics belonging to the carbapenem class (meropenem, ertapenem and imipenem), our bacterial isolates exhibited resistance rates ranging from 42% to 50%. Among all *K. pneumoniae* isolates investigated, 31 (62%) were classified as MDR and 19 (38%) as XDR. These classifications were determined based on the full antimicrobial panel tested in this study, covering major antibiotic classes relevant to clinical *K. pneumoniae* infections.

### 3.3. Molecular Investigations of Carbapenemase-Encoding Genes

Molecular investigations of carbapenemase-encoding genes were performed using multiplex PCR. For assay validation, positive-control DNA corresponding to each target carbapenemase gene and a no-template negative control were included to verify primer performance and the expected amplicon sizes. Isolates were then analyzed under the same PCR conditions and amplification products were resolved together on the same agarose gel to facilitate direct comparison of the detected carbapenemase genes (Supplementary Figure S2).

Among a total of 50 *K. pneumoniae* isolates, multiplex PCR amplifications revealed that the *blaOXA-48* gene was detected in 28 isolates (56%), *blaIMP* in 22 isolates (44%) (IMP-1 within 12 isolates and IMP-2 within 10 isolates), *blaVIM* in 15 isolates (30%), *blaKPC* in 14 isolates (28%) and *blaNDM* in 13 isolates (26%). Notably, the IMP-8 variant was identified in two isolates using the IMP-2 specific primers. DNA sequencing enabled the identification of the IMP-8 variant, as closely related variants with high genetic similarity produce PCR amplicons of the same expected size. Furthermore, 60% (30/50) of *K. pneumoniae* isolates carried multiple carbapenemase genes. Notably, four isolates co-harboured four genes while ten carried three genes simultaneously (Supplementary Figure S2).

In this study, 12 strains of *Klebsiella pneumoniae* were analyzed to identify mutations within antibiotic resistance genes. In fact, sequencing was performed on two representative PCR-positive isolates for each detected carbapenemase gene to provide preliminary mutation screening, resulting in a total of 12 sequences. DNA sequence alignments with close homologs from NCBI allowed us to detect several point mutations (varying from 0 to 4 mutations per gene) (Supplementary Table S1). While some of these substitutions were located in proximity to the active sites of the enzymes, most mutations were found at distant locations. The translated amino acid sequences, further aligned to reference proteins, revealed the presence of point mutations in 11 sequences among 12. Table S2 in Supplementary Materials summarizes DNA mutations, amino acid changes and their structural location along with their predicted effect on the protein. Supplementary Tables S3 and S4 present the detailed in silico analysis scores generated with the computational predictive tools.

Molecular docking analyses were further conducted to study whether the amino acid substitutions could affect the interactions between the enzyme and its substrate (carbapenem antibiotic) through binding affinity measurements. Meropenem was docked against wild-type and mutant carbapenemase proteins (IMP-1 and 2, KPC, NDM, OXA-48, and VIM) and results showed that the binding affinities were almost identical for each carbapenemase (Table 3). For instance, the binding energy of the substrate to IMP-2, VIM-1, KPC-2 and OXA-48 wild types and their mutants exhibited the same binding energies. Similarly, IMP-1 and NDM-52 wild-type and mutant proteins showed minimal difference in binding energies (Table 3). In this in silico analysis, no major differences were observed, which suggests that the amino acid substitutions did not significantly alter the binding affinity of meropenem.

## 4. Discussion

From a total of 256 clinical samples collected during the study period (from October 2021 to February 2022), fifty isolates of *Klebsiella pneumoniae* were recovered, mainly from sputum and urine samples, with an overall frequency of 19.53%. Although bacterial identification was supported by 16S rRNA gene amplification, it should be noted that the 16S rRNA gene has limited discriminatory power within the *Klebsiella pneumoniae* species complex due to the high genetic similarity among closely related taxa.

In a recent Iraqi review, *Klebsiella pneumoniae* was identified as the most prevalent Gram-negative bacteria (GNB), accounting for 0% [30]. In addition, isolation rates from urine, blood and sputum broadly match findings from studies conducted in Iraq, from Al-Diwaniyah city [31], Erbil [32], Duhok [33], Anbar province [34] and Baghdad [35,36], as well as from studies conducted elsewhere in the world [37,38,39]. Overall, our results and those reported in previous studies [9,40,41] support the finding that *K. pneumoniae* has the ability to cause a wide spectrum of clinical illness and can be isolated from a variety of clinical specimens. *Klebsiella pneumoniae* infections are reported worldwide and represent a global public health threat that requires effective actions, especially in developing countries such as Iraq where surveillance systems remain limited [42,43].

*K. pneumoniae* isolates exhibited high resistance rates to penicillins and cephalosporins, ranging from 80 to 90%, similarly to results observed across other cities in Iraq [33,34,44,45,46]. A large proportion of the isolates (80%) exhibited resistance to the fourth-generation cephalosporin cefepime, consistent with findings from Iraqi studies by Mohanna and AL-Yasseen in 2024 (90%) [45], Majid and Zaki in 2022 (85%) [46], Al-Qaysi et al. in 2024 (75%) [34] and Naqid et al. in 2020 (60%) [33]. In fact, resistance to penicillins and cephalosporins may be due to the production of β-lactamases and carbapenemases, as well as mutations in penicillin-binding proteins (PBPs), decreased outer membrane permeability and active efflux [47,48]. Some β-lactamases are characterized by their ability to rapidly mutate and expand their spectrum of activity; they have considerable activity against penicillins, early cephalosporins and even imipenem [49]. For fluoroquinolones, 58% of the isolates exhibited decreased susceptibility to ciprofloxacin. Similar and higher percentages were reported in Iraqi studies by Al-Qaysi et al. in 2024 [34] and Mohanna and AL-Yasseen in 2024 [45], respectively, whereas a lower percentage was observed by Al Meani and Ahmed in 2019 [44]. Isolates recovered in this study showed resistance rates ranging from 42% to 50% for carbapenem antibiotics. These rates are higher than those reported in Duhok City by Naqid et al. in 2020 [33] and in Anbar by Al Meani and Ahmedin 2019 [44]. Multidrug resistance was observed within 62% of *K. pneumoniae* strains, while 38% were characterized as XDR. This represents the first report of such data in Al-Diwaniyah city/Iraq. In other cities, MDR rates of 33% were reported in Anbar province [34], 82% in Baghdad [36] and 44.4% in Najaf [45]. The proportion of MDR *K. pneumoniae* observed in this study, as well as in other reports [50,51,52], is among the highest reported in the literature. Variations in MDR rates can be attributed to differences in geographical location, sanitation levels and antibiotic prescribing practices. Our study emphasizes the overall alarming rates of antibiotic resistance among clinical *K. pneumoniae*, which highlights the urgent need for a continuous surveillance system for bacterial antibiotic resistance. Future investigations incorporating quantitative susceptibility methods and phenotypic carbapenemase confirmation assays would provide a more comprehensive characterization of resistance profiles.

All *K. pneumoniae* isolates carried at least one carbapenemase gene, with 60% co-harbouring multiple genes. Carbapenemase genes were detected as follows: *blaOXA*-*48* (56%), *blaIMP* (44%), *blaVIM* (30%), *blaKPC* (28%,) and *blaNDM* (26%). Similarly to our results, the highest rates of carbapenem genes were for the *blaOXA* gene, followed by *blaVIM* and *blaNDM*, according to several national studies from various cities [25]. Similar findings were reported in Baghdad by Ahmed in 2022 [24], who observed detection rates of 38% for *blaIMP* and 36% for each of *blaOXA-48* and *blaVIM*, followed by *blaKPC* (21%) and *blaNDM* (13%) [24], and Hamad et al. in 2022 [53], who detected *blaIMP* in 37.5% of *K. pneumoniae*, followed by *blaOXA-48*: 30%, *blaVIM*: 28.75%, *blaKPC*: 20%, and *blaNDM*: 11.25%. Our results are also similar to several Iraqi studies reporting a relatively high frequency of *OXA-48* and *blaIMP* genes in different clinical pathogens [25]. Nevertheless, our findings disagree with those reported in Najaf by Mohanna and AL-Yasseen in 2024 [45], who reported that neither *blaOXA-48* nor *blaKPC* genes were found. As demonstrated by Hasan et al. in 2022 [25], Iraqi rates may vary by province, period and method of detection. In fact, the prevalence of *blaOXA-48*, described for the first time in 2001 in Turkey within a *Klebsiella pneumoniae* isolate [54], is emerging and rapidly disseminating within *Enterobacterales* across Europe, North Africa, and the Middle East [54,55]. Its detection in *Klebsiella pneumoniae* is rising globally, with reported rates as high as 90% [56,57,58,59,60].

Furthermore, we hereby report that 30 *K. pneumoniae* isolates (60%) carried multiple carbapenemase genes. Mohanna and AL-Yasseen [45] reported a percentage of 80% in Najaf in 2024. Notably, four isolates from our study co-harboured four genes while ten carried three genes simultaneously (Supplementary Figure S2). Thus, the frequent co-occurrence of multiple carbapenemase genes within the same isolates and the relatively limited number of isolates limited the ability to perform meaningful genotype–phenotype correlation analyses.

The discrepancy between the presence of carbapenemase-encoding genes and the observed phenotypic resistance (42–50%) highlights the complexity of genotype–phenotype relationships in *K. pneumoniae*. The presence of carbapenemase genes does not always directly translate into phenotypic resistance, as gene expression levels, regulatory mechanisms, enzyme activity, and additional resistance determinants such as outer membrane permeability changes and efflux pump activity can significantly influence the resistance phenotype. Moreover, the genetic background of individual isolates and other biological factors may contribute to differences in carbapenem resistance expression. The interpretation of carbapenemase-related findings should also consider that phenotypic confirmation of carbapenemase production was not performed in this study. While molecular detection of carbapenemase-encoding genes provides important evidence of resistance-associated genetic determinants, gene carriage alone does not necessarily reflect their expression level or enzymatic activity. Phenotypic confirmation assays, such as the modified carbapenem inactivation method (mCIM/eCIM) or Carba NP test, were not performed and represent an important area for future investigation to further validate the functional contribution of the detected carbapenemase genes.

Our observations are consistent with recent studies highlighting the alarming capacity of hospital-associated bacterial strains to harbour multiple resistance genes simultaneously, which compromises therapy and limits treatment options [61]. The extensive use of antibiotics, the lack of antimicrobial resistance surveillance programs, and insufficient control procedures in hospital settings may all contribute to the high levels of antimicrobial resistance reported in this study. Additionally, the spread of MDR *K. pneumoniae* strains is accelerated by the presence of resistance determinants on mobile genetic elements. These findings are consistent with previous reports indicating an increasing burden of antimicrobial resistance in low- and middle-income countries and emphasize the need for strengthened surveillance and control strategies.

The structural and functional evaluation of the studied mutations revealed some molecular features that may influence enzyme structure and stability (Supplementary Table S2). A limited number of sequenced isolates was analyzed in this study as a preliminary investigation. Therefore, the present sequencing data should not be interpreted as representative of the overall diversity or prevalence of carbapenemase variants among the studied isolates. Accordingly, the results further discussed are interpreted as exploratory and hypothesis-generating.

The IMP-1 carbapenemase is a class B metallo-β-lactamase with a zinc-dependent catalytic mechanism for the hydrolysis of β-lactam antibiotics. For this enzyme, the mutation in isolate 1 (N141Y) is located near the flexible L3 loop of the protein. This region is involved in shaping the substrate-binding site. The substitution introduces a larger aromatic residue that could potentially influence local structural features, such as loop flexibility. However, the impact of this change on enzymatic activity cannot be conclusively determined based solely on computational analyses. The observed structural differences suggest a possible role in modulating the local environment of the enzyme, but experimental studies are required to assess whether this substitution affects β-lactam hydrolysis or overall enzyme function.

Within the IMP-8 carbapenemase, the P113H substitution detected in both isolates is noteworthy because the P113 residue is located close to the zinc-binding site. The replacement by histidine, a residue capable of metal coordination, may alter the local chemical environment surrounding the zinc-binding region. However, whether this substitution affects zinc coordination or enzymatic stability cannot be established from computational analysis alone. For isolate 2, the mutation Y63N lies close to the outer surface, near the loops contributing to substrate recognition. Computational analysis suggested that this substitution may affect local loop characteristics, which could potentially influence the structural environment involved in substrate recognition. However, whether this change translates into altered substrate binding or catalytic properties requires experimental validation. Similar studies have reported that single amino acid substitutions located near the active site of IMP enzymes may influence substrate interactions and resistance profiles, as observed for xeruborbactam resistance-associated variants in IMP-6 and IMP-10 [62]. These findings highlight the potential contribution of individual substitutions to enzyme adaptability, although the specific functional role of the mutation identified in the present study remains to be determined.

In fact, genetic variations within metallo-β-lactamases have been reported to be associated with phenotypic variability related to antibiotic resistance [63]. Overall, MBLs, such as IMP enzymes, can accumulate numerous variants under selective pressure. Such substitutions may influence structural features and interactions with β-lactam substrates, potentially contributing to variations in substrate profiles and enzymatic properties, consistent with the evolutionary trade-off in substrate specificity described for β-lactamases [64].

The structural evaluation of the VIM-1 metallo-β-lactamase, a class B enzyme reliant on zinc coordination for β-lactam hydrolysis, demonstrated that in both isolates, the single mutation A97T occurs on a surface loop outside the active site. The conservative nature of this substitution suggests limited structural consequences. Both strains preserve the structural integrity of the active site. Previous observations across the VIM enzymes align with our finding regarding mutations near peripheral loops, which are tolerated or neutral [65,66,67].

The evaluation of amino acid substitutions in NDM-52 β-lactamase revealed two mutations in isolate 1, namely T173N and H120L. While T173N is predicted to have limited structural impacts, H120L occurs at a residue reported to participate in Zn1 coordination within the catalytic site. Replacement of histidine by leucine may modify the local zinc-coordination environment and active-site geometry, as leucine lacks metal-coordinating properties. Thus, this substitution could potentially influence enzyme stability or catalytic properties; however, its actual impact on enzyme function requires experimental validation.

Previous studies have shown that amino acid substitutions occurring in or near the active site of metallo-β-lactamases may influence substrate recognition and resistance phenotypes [68,69]. NDM-8 and NDM-9 variants carrying substitutions close to the catalytic region have been reported to exhibit modified activity against certain β-lactam antibiotics, including ceftazidime and cefiderocol [70,71].

The class A carbapenemase KPC-2 is a serine β-lactamase with a catalytic triad and a flexible Ω-loop that defines the geometry and dynamics of the active site. Both clinical isolates harbour a single mutation: I107F. This mutation is not located directly within the catalytic site and, based on computational analysis, does not appear to induce major structural alterations.

OXA-48 is a class D serine β-lactamase with a narrow active-site groove. In isolate 1, the substitution N106K is located near the β5–β6 loop adjacent to the active site. This region is involved in the shaping of the substrate-binding pocket. However, computational analysis suggests that the mutation does not induce major changes in the active-site configuration and may be structurally tolerated. In isolate 2, the mutation V120L may be of greater structural interest. Located at the entrance of the substrate-binding site, V120L could potentially contribute to variations in the catalytic environment; however, its impact on enzyme fitness or resistance phenotype remains to be experimentally determined. Interestingly, previous studies have suggested that such substitutions may enhance local flexibility, which could represent an adaptive mechanism in these enzymes [72,73,74]. According to Dabos et al., 2020 [75], such modifications were marked by an increase in expanded-spectrum cephalosporin activity, potentially through structural changes affecting substrate accommodation. In the same context, Fröhlich et al. in 2022 [76] demonstrated that one or two mutations in the OXA-48 wild type were associated with increased cefiderocol resistance, highlighting the capacity of these enzymes to adapt under selective pressure.

Because the computational tools employed in this study evaluate different aspects of mutation effects, occasional differences among their predictions are expected. DDMut and FiTMuSiC estimate changes in protein thermodynamic stability using distinct computational frameworks, whereas SIFT and PROVEAN predict the potential functional impact of amino acid substitutions primarily from evolutionary sequence conservation rather than direct measurements of enzymatic activity. Moreover, small differences in predicted stability may be classified differently by individual algorithms because they rely on distinct computational models and scoring functions. Consequently, discordant classifications between these approaches do not necessarily represent contradictory results but rather reflect complementary perspectives on the potential structural and functional consequences of the mutations. Therefore, the computational predictions were interpreted collectively as hypothesis-generating evidence rather than definitive indicators of altered enzyme activity.

Overall, mutations within carbapenemase enzymes may have variable functional consequences depending on their location within the enzyme and their proximity to substrate-binding regions. The above-discussed findings highlight the complex relationship between natural carbapenemase polymorphisms and their potential functional consequences, with some variants having limited effects, while others may contribute to broader substrate profiles or subtle changes in resistance phenotypes under selective pressure [76,77]. However, given the limited number of sequenced isolates analyzed in this study, further investigations involving larger sequencing datasets are required to better characterize the diversity of carbapenemase variants and to confirm the structural and functional significance of the identified substitutions.

**Figure 1:**
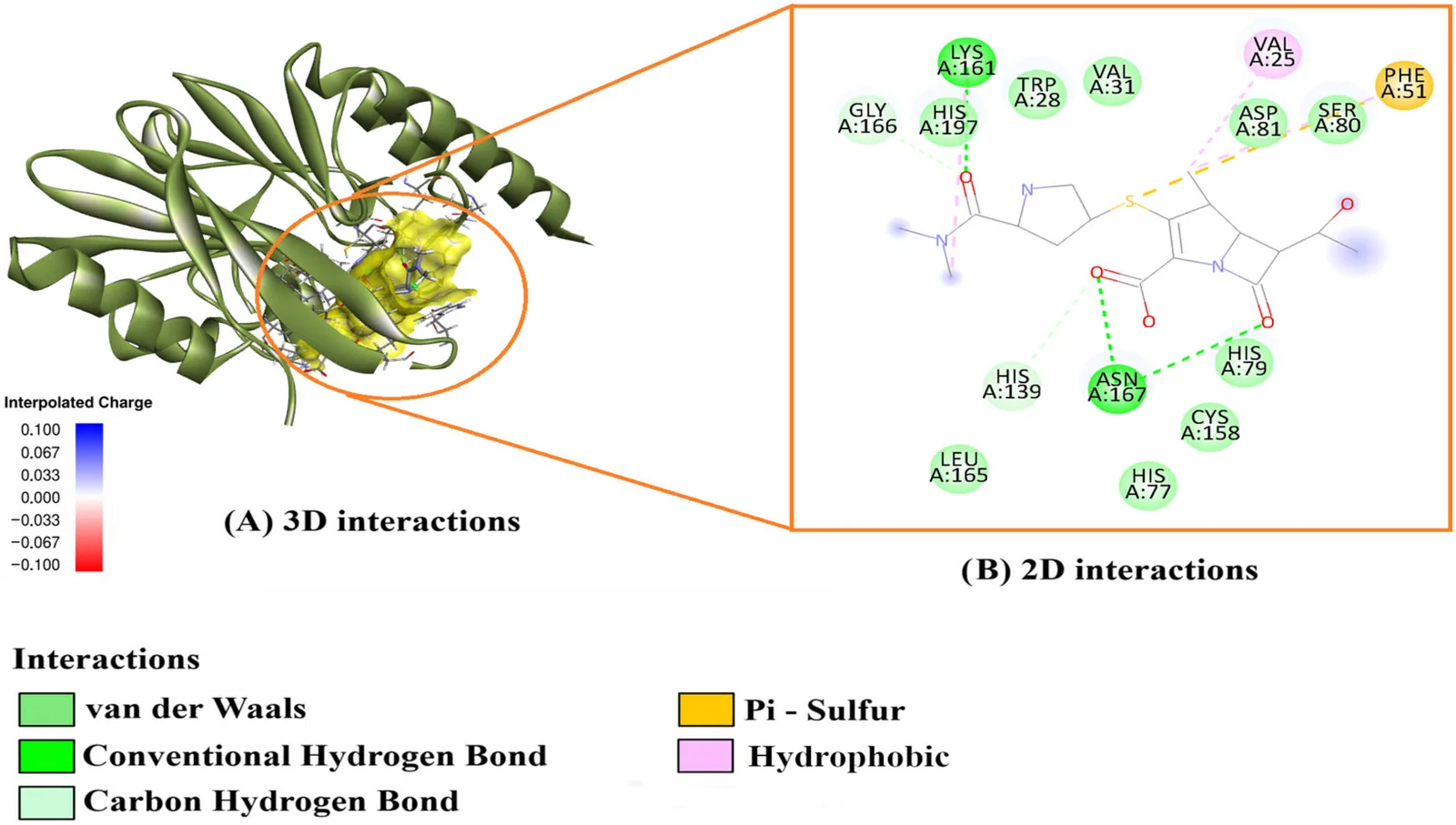
(**A**) Three-dimensional representation of the meropenem–IMP-1-wildtype complex illustrating molecular interactions; (**B**) two-dimensional diagram highlighting the principal binding residues and interaction types.

**Figure 2:**
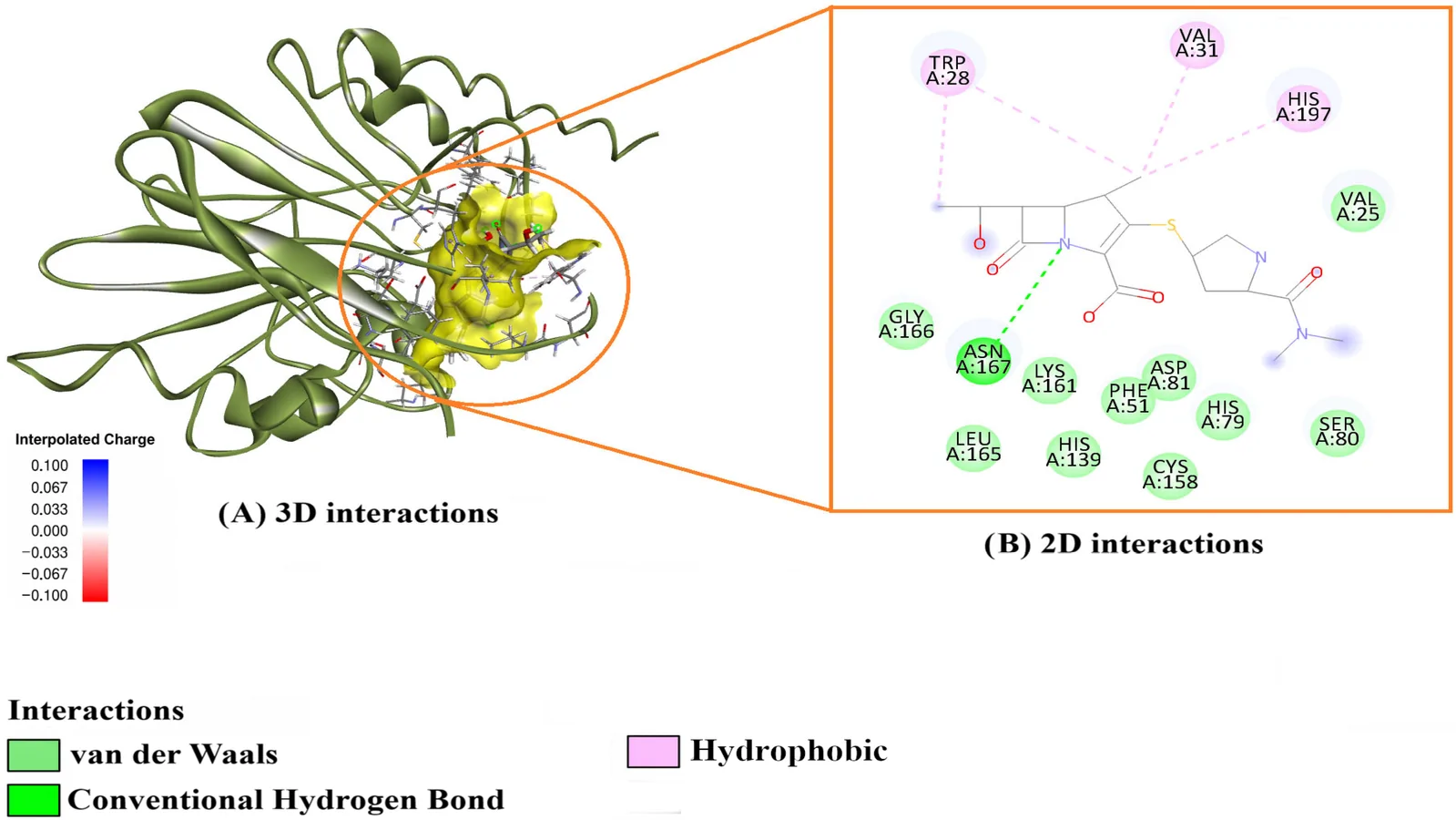
(**A**) Three-dimensional representation of the meropenem–IMP-1-mutant1 complex illustrating molecular interactions; (**B**) two-dimensional diagram highlighting the principal binding residues and interaction types.

**Figure 3:**
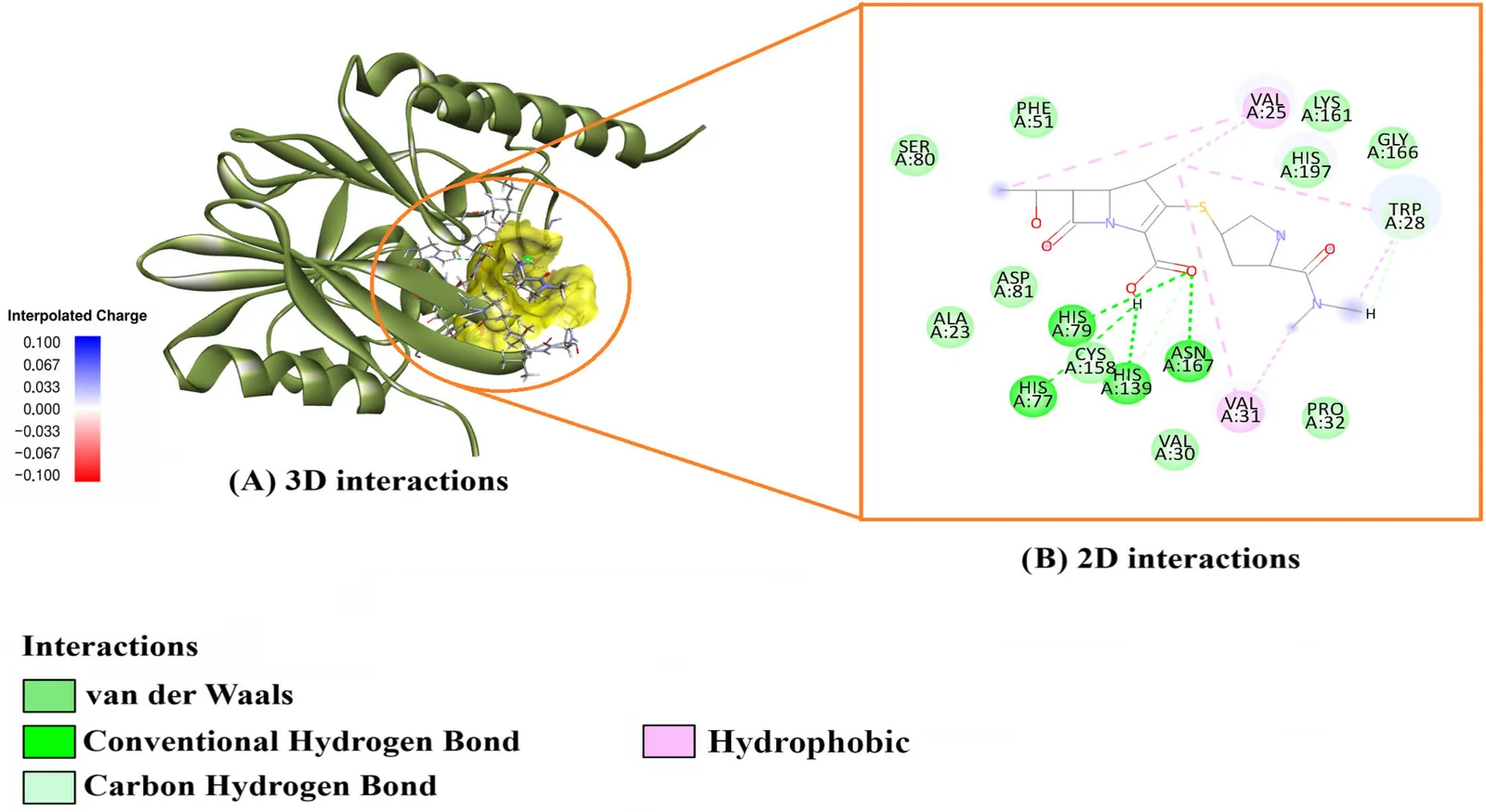
(**A**) Three-dimensional representation of the meropenem–IMP-1-mutant2 complex illustrating molecular interactions; (**B**) two-dimensional diagram highlighting the principal binding residues and interaction types.

In order to investigate whether point mutations could affect enzyme–substrate interactions, molecular docking was performed to estimate the predicted binding affinities of the substrate (meropenem) within the active site of each enzyme (Figure 1, Figure 2 and Figure 3). Meropenem was selected as the docking ligand because it is a clinically relevant carbapenem widely used in the management of infections caused by carbapenem-resistant Enterobacterales and represents a commonly investigated substrate for carbapenemases. Although different carbapenemases may display variable substrate preferences, the docking analysis with meropenem provides valuable comparative insights into potential mutation-associated changes in enzyme–substrate interactions.

Molecular docking analyses indicated that the identified amino acid substitutions did not produce meaningful changes in the predicted binding affinity of meropenem toward the investigated carbapenemases, as only minimal differences in binding energies were observed between the wild-type and mutant proteins. Therefore, the docking results do not provide evidence that these substitutions significantly alter enzyme–substrate binding under the conditions of this in silico analysis. These findings should be considered preliminary and exploratory, and their biological relevance requires further validation through in vitro experimental studies. Although some complementary in silico predictions suggested possible structural or stability effects for certain substitutions, the effects of the studied mutations on protein stability or catalytic efficiency cannot be excluded and require further investigation. Further studies, including enzymatic assays and molecular dynamics simulations, are required to determine the biological significance of these mutations.

## 5. Conclusions

The current study showed a high prevalence of clinical multidrug-resistant *Klebsiella pneumoniae* isolates in Al-Diwaniyah, Iraq, with widespread resistance to different antibiotic classes, including carbapenems. The identification of diverse carbapenemase genes, particularly *blaOXA-48*, alongside multiple gene co-carriages in a single strain, highlights the complexity and severity of antimicrobial resistance. Molecular and structural analyses suggested that, although many identified amino acid substitutions are likely functionally tolerated, specific changes such as H120L in NDM-52 and V120L in OXA-48, located near catalytic or substrate-interacting regions, may influence local structural features; however, their biological significance remains to be experimentally determined. Molecular docking indicated that these mutations preserved predicted meropenem binding affinity, suggesting that differences among carbapenemase variants may involve factors beyond direct substrate-binding interactions. Overall, these findings contribute to the understanding of carbapenem resistance diversity in *K. pneumoniae* and emphasize the critical need for implementing efficient antimicrobial resistance surveillance programs to limit the dissemination of highly resistant *K. pneumoniae* in Iraq.

### Limitations

Since the study was designed primarily for phenotypic and molecular characterization rather than epidemiological investigation, analyses focused on overall trends among the studied isolates and isolate-level metadata were not presented. Despite providing important insights into the molecular basis of carbapenem resistance in *Klebsiella pneumoniae*, the limited number of sequenced isolates per gene makes the interpretation of the mutation analysis preliminary and exploratory.

The functional and structural impacts of the detected mutations were studied using in silico tools, without experimental confirmation. Molecular docking analyses indicated that the studied amino acid substitutions did not significantly alter the predicted binding affinity of meropenem. However, these docking results alone cannot be used as evidence that the detected mutations produce functional changes in substrate binding or catalytic activity. Functional effects inferred from sequence- or structure-based predictions should therefore be considered exploratory and require further experimental validation.

## Figures and Tables

**Table 1:** PCR primers for carbapenemase antibiotic resistance genes and their amplicon sizes.

Target Gene	Primer Sequences (5′–3′)		Product Size (bp)
*blaKPC*	F	ATGTCACTGTATCGCCGTCT	520
	R	TTTTCAGAGCCTTACTGCCC	
*blaIMP1*	F	TGAGCAAGTTATCTGTATTC	600
	R	TTAGTTGCTTGGTTTTGATG	
*blaIMP2*	F	GGCAGTCGCCCTAAAACAAA	406
	R	TAGTTACTTGGCTGTGATGG	
*blaVIM*	F	GATGGTGTTTGGTCGCATA	475
	R	CGAATGCGCAGCACCAG	
*blaNDM*	F	GGTTTGGCGATCTGGTTTTC	314
	R	CGGAATGGCTCATCACGATC	
*blaOXA-48*	F	TTGGTGGCATCGATTATCGG	725
	R	GAGCACTTCTTTTGTGATGGC	

F: forward. R: reverse.

**Table 2:** Antimicrobial susceptibility and resistance rates of clinical *K. pneumoniae* isolates.

Antibiotic Class	Antibiotic	Resistant *n* (%)	Intermediate *n* (%)	Susceptible (%)
β-lactams-Penicillins	ampicillin (AMP)	50 (100%)	0 (0%)	0 (0%)
β-lactams-Penicillin + β-lactamase inhibitor	piperacillin–tazobactum (TPZ)	38 (76%)	2 (4%)	10 (20%)
	amoxicillin/clavulanic acid (AMO)	50 (100%)	0 (0%)	0 (0%)
β-lactams-Extended spectrum penicillins	temocillin (TEM)	47 (94%)	3 (6%)	0 (0%)
β-lactams-Cephalosporins	cefuroxime (CXM)	45 (90%)	3 (6%)	2 (4%)
	cefoxitin (FOX)	45 (90%)	0 (0%)	5 (10%)
	cefepime (CPM)	40 (80%)	0 (0%)	10 (20%)
	ceftriaxone (CRO)	48 (96%)	0 (0%)	2 (4%)
	ceftazidime (CAZ)	45 (90%)	0 (0%)	5 (10%)
	cefotaxime (CTX)	50 (100%)	0 (0%)	0 (0%)
Fluoroquinolones	ciprofloxacin (CIP)	29 (58%)	2 (4%)	19 (38%)
Carbapenems	ertapenem (ERT)	24 (48%)	5 (10%)	21 (42%)
	imipenem (IPM)	21 (42%)	0 (0%)	29 (58%)
	meropenem (MEM)	25 (50%)	2 (4%)	23 (46%)

*n* = number of isolates.

**Table 3:** Docking results of meropenem against wild-type and mutant (m1/m2) carbapenemase enzymes.

Protein	Binding Energy (kcal/mol)	LE	FQ	BEI	Ki (μM)
IMP-1	−7.6	0.292	0.695	0.019	2.660
IMP-1m1	−7.6	0.292	0.695	0.019	2.660
IMP-1m2	−7.9	0.304	0.724	0.020	1.600
IMP-2	−7.2	0.277	0.660	0.018	5.210
IMP-2m1	−7.2	0.277	0.660	0.018	5.210
IMP-2m2	−7.2	0.277	0.660	0.018	5.210
VIM-1	−7.7	0.296	0.705	0.020	2.240
VIM-1m1	−7.7	0.296	0.705	0.020	2.240
VIM-1m2	−7.7	0.296	0.705	0.020	2.240
KPC-2	−7.7	0.296	0.705	0.020	2.240
KPC-2m1	−7.7	0.296	0.705	0.020	2.240
KPC-2m2	−7.7	0.296	0.705	0.020	2.240
NDM-52	−7.0	0.269	0.640	0.018	7.350
NDM-52m1	−6.9	0.265	0.631	0.018	8.730
OXA-48	−7.2	0.277	0.660	0.018	5.210
OXA-48m1	−7.2	0.277	0.660	0.018	5.210
OXA-48m2	−7.2	0.277	0.660	0.018	5.210

BEI: Binding Efficiency Index, FQ: Fit Quality, Ki: Estimated Inhibition Constant, LE: Ligand Efficiency.

## Data Availability

The original contributions presented in this study are included in the article/Supplementary Materials. Further inquiries can be directed to the corresponding authors.

## Supplementary Materials

The following supporting information can be downloaded at: https://www.mdpi.com/article/10.3390/pathogens15080819/s1, Table S1: Analysis of carbapenemase gene mutations compared to the close homologs retrieved from NCBI and amino acid changes in the corresponding proteins; Table S2: Predicted functional impacts of point mutations in carbapenemase enzymes across clinical isolates of *K. pneumoniae*; Table S3: Computational predictions (SIFT, PROVEAN, DDMut) of mutational effects on carbapenemase enzymes from clinical MDR *K. pneumoniae;* Table S4: FiTMuSiC analysis of structural and functional effects of mutations on carbapenemase enzymes from clinical MDR *K. pneumoniae;* Figure S1: Agarose gel electrophoresis of PCR-amplified 16S ribosomal RNA gene from *Klebsiella pneumoniae* isolates showing the expected 551 bp product (Marker ladder 2000–100 bp). (A) PCR-positive isolates, lanes 1–37; (B) PCR-positive isolates, lanes 38–50; Figure S2: Agarose gel electrophoresis of PCR-amplified carbapenemase genes in *Klebsiella pneumoniae* isolates. The targeted genes and their expected sizes are: bla-KPC, 520 bp; bla-IMP1, 600 bp; bla-IMP2, 406 bp; bla-VIM, 475 bp; bla-NDM, 314 bp; and bla-OXA-48, 725 bp. M: DNA ladder (2000–100 bp).
